# Is Rheumatoid Arthritis Related to Coffee Consumption in Korea? A Nationwide Cross-Sectional Observational Study

**DOI:** 10.3390/ijerph18157880

**Published:** 2021-07-25

**Authors:** Sang-Gyun Kim, Jong Woo Kang, Seong Min Jeong, Gwan Gyu Song, Sung Jae Choi, Jae Hyun Jung

**Affiliations:** 1 National Medical Center, Department of Orthopedic Surgery, 245 Eulji-ro, Jung-gu, Seoul 04564, Korea; mup81@hotmail.com (S.-G.K.); inplate@nmc.or.kr (S.M.J.); 2 Department of Medicine, Korea University College of Medicine, 73 Goryeodae-ro, Seongbuk-gu, Seoul 02841, Korea; oskang@korea.ac.kr (J.W.K.); gsong@kumc.or.kr (G.G.S.); csjmd888@korea.ac.kr (S.J.C.); 3 Department of Orthopedic Surgery, Korea University Ansan Hospital 123 Jeokgeum-ro, Danwon-gu, Ansan-si 15355, Gyeonggi-do, Korea; 4 Department of Internal Medicine, Division of Rheumatology, Korea University Guro Hospital, 148 Gurodong-ro, Guro-gu, Seoul 08308, Korea; 5 Department of Internal Medicine, Division of Rheumatology, Korea University Ansan Hospital, 123 Jeokgeum-ro, Danwon-gu, Ansan-si 15355, Gyeonggi-do, Korea

**Keywords:** coffee, rheumatoid arthritis, prevalence

## Abstract

Coffee consumption is gradually increasing in Korea. As a result, interest in the relationship between coffee consumption and various diseases is growing. Several factors affect the development of rheumatoid arthritis (RA), and coffee consumption may be related. We conducted a nationwide cross-sectional study using data from the Korea National Health and Nutrition Examination Survey (2012–2016). A total of 12,465 eligible participants (4819 men and 7646 women) were included in the study. Participants with RA were defined as those who were diagnosed and currently being treated by physicians. Daily coffee consumption amounts were categorized as none, <1 cup, 1–2 cups, 2–3 cups, and ≥3 cups a day based on a self-report. A multivariable logistic regression model was employed, and we calculated the odds ratios (ORs) and 95% confidence intervals (CIs) for the odds of participants having RA with respect to coffee consumption. Compared to the no-coffee group, the ORs for RA in the <1 cup and 1–2 cups groups were 2.99 (95% CI 0.33–27.28) and 2.63 (95% CI 0.31–22.63) in men, respectively, and the ORs for RA for women in the <1 cup, 1–2 cups, 2–3 cups, and ≥3 cups groups were 0.62 (95% CI 0.31–1.26), 0.67 (95% CI 0.33–1.37), 1.08 (95% CI 0.35–3.36), and 1.43 (95% CI 0.25–8.36), respectively. Our study concludes, therefore, that daily coffee consumption is not related to the prevalence of RA in the general Korean population.

## 1. Introduction

Rheumatoid arthritis (RA) is a chronic inflammatory musculoskeletal disease characterized by synovitis of multiple joints causing swelling, stiffness, pain, and progressive destruction, which has a worldwide prevalence of 0.5–1.0% in adults [1]. Both genetic and environmental factors play a role in the development and progression of RA. Smoking is a representative environmental factor involved in the development of RA [1,2]. Moreover, it has been suggested that not only smoking, but various dietary factors are also associated with the development RA [3].

Coffee is one of the most commonly consumed beverages worldwide. Coffee consumption in Korea is continuously increasing, and Koreans drink approximately 12 cups of coffee per week [4]. The health effects of coffee consumption have become of increasing interest. Coffee has a protective effect against cardiovascular disease and diabetes, while it increases the risk, or shows controversial findings, with respect to musculoskeletal diseases such as osteoarthritis, osteoporosis, and hip fracture [5,6]. Coffee is believed to have a protective effect against the development and progression of RA by reducing T cell activation, B cell proliferation, tumor necrosis factor (TNF)-α release, and increasing interleukin (IL)-10 release [7]. Previous studies have investigated the relationship between coffee consumption and RA; however, these studies were conducted with different study designs, and have shown conflicting results [8,9,10]. Furthermore, most previous studies on the association between coffee consumption and RA have been performed in predominantly white populations. To the best of our knowledge, there has been no studies on the relationship between coffee consumption and RA in Asians populations.

The prevalence of RA varies according to ethnicity, and coffee consumption patterns differ by geographic region [11]. In Korea, the prevalence of both RA and coffee consumption is increasing; thus, an investigation into whether coffee consumption is related to RA is believed to be a meaningful study for diet management in patients with RA [6,12]. This study investigates the relationship between coffee consumption and RA in the Korean population using nationwide data spanning five years (2012–2016) from the Korea National Health and Nutrition Examination Survey (KNHANES).

## 2. Materials and Methods

### 2.1. Study Design and Setting

This study was cross-sectional in design and used the KNHANES, which collected data for five years, between 2012 and 2016. The KNHANES is a nationwide survey administered by the Korea Centers for Disease Control and Prevention (KCDC), and the institutional review board of the KCDC gave its approval for our study [13]. The study was conducted in accordance with the Helsinki Declaration of 2000. Among the subjects selected through proportional systemic sampling, those who provided informed consent participated in the survey. Households were randomly selected for participation and sampled by multistage stratification based on geographical areas. The informed consent form given to each participant can be viewed in the KCDC database. The requirement for informed consent for this study was waived due to the fact that it performs secondary analysis based on a pre-existing KNHANES dataset.

### 2.2. Participants

In the 2012–2016 KNHANES, 12,465 participants were surveyed for both coffee consumption and RA, including 4819 men and 7646 women.

### 2.3. Main Variables and Covariates

The RA group included participants diagnosed with RA by a physician and who were currently taking medications for RA. Among the participants who responded to questionnaires about RA, those who did not have RA were defined as non-RA participants. Participants were asked how many cups of coffee they drink per day, week, and month. A cup of coffee was defined as the amount of coffee containing two teaspoons of coffee grounds. The average caffeine content in a cup of the 10 most frequently consumed coffee brands in Korea was 48.96 ± 7.41 mg [6]. Based on coffee consumption, participants were divided into five groups: (1) do not drink coffee, (2) drink <1 cup of coffee per day, (3) drink 1–2 cups of coffee per day, (4) drink 2–3 cups of coffee per day, and (5) drink ≥3 cups of coffee per day.

Sex, age, body mass index (BMI), hypertension (HTN), diabetes mellitus (DM), dyslipidemia, alcohol consumption, smoking status, household income, and education level were considered as potential confounding variables affecting RA risk and coffee consumption. BMI was calculated as weight (kg) divided by height squared (m^2^). HTN was defined as an average systolic blood pressure ≥140 mmHg, diastolic blood pressure ≥90 mmHg, or use of antihypertensive medications. Prehypertension was defined as a blood pressure ≥120/80 mmHg. DM was defined as a fasting plasma glucose (FPG) level ≥126 mg/dL, diagnosis of DM by a clinician, or use of an oral hypoglycemic agent or injected insulin. Dyslipidemia was defined as follows: total cholesterol ≥200 mg/dL, triglyceride ≥150 mg/dL, high-density lipoprotein cholesterol <40 mg/dL in men and <50 mg/dL in women, or currently taking any anti-dyslipidemic drugs for controlling blood lipid concentrations. Data regarding alcohol consumption, smoking status, household income, and education level were collected during the health interviews. The participants were classified according to alcohol consumption as follows: heavy drinkers who consumed an average of ≥7 units of alcohol for men and ≥5 units for women ≥2 days per week, moderate drinkers who consumed more than one glass of alcohol per month over the past year, and nondrinkers who never drink or drank less than one unit of alcohol per month over the past year. One unit refers to one glass, which contains approximately 10 mL of alcohol. The smoking status groups comprised of current, former, and non-smokers. Monthly household income was categorized into quartiles: lowest (USD < 918.3), lower middle (USD 918.3–1836.6), upper middle (USD 1836.6–3213.9), and highest (USD > 3213.9). Education level was classified as primary school or lower, middle school, high school, and university or higher.

### 2.4. Statistical Analysis

The odds ratios (ORs) and 95% confidence intervals (95% CIs) for RA according to coffee consumption were calculated. Sex was stratified and four different logistic regression models were used to assess the association between coffee consumption and RA. Model I was adjusted for age and BMI; model II was adjusted for age, BMI, HTN, DM, and dyslipidemia; model III was adjusted for age, BMI, HTN, DM, dyslipidemia, alcohol consumption, and smoking status; and model IV was adjusted for age, BMI, HTN, DM, dyslipidemia, alcohol consumption, smoking status, household income, and education level. The *p*-values for trends in RA according to coffee consumption were calculated using the Cochran-Armitage trend test. The multiple imputation analysis was performed for the missing data. SPSS ver. 23.0 (SPSS Inc., Chicago, IL, USA) was used for all statistical analyses, and a *p*-value < 0.05 was considered to indicate significance.

## 3. Results

### 3.1. Baseline Characteristics

The prevalence of RA among participants in this study was 0.5% for men, 0.9% for women, and 0.8% overall. For both men and women, participants with RA were older and had lower education levels than those without RA; however, there were no significant differences in BMI, DM, dyslipidemia, alcohol consumption, smoking status, or coffee consumption. In women, the prevalence of HTN was higher in the RA group than in the non-RA group, whereas in men, the income level was lower in the RA group than in the non-RA group. The baseline characteristics of the participants with and without RA are summarized in Table 1.

The demographic characteristics of the participants according to coffee consumption are summarized in Table 2. In both men and women, the higher the consumption of coffee, the higher the percentage of drinkers and smokers, and the higher the household income and education level.

### 3.2. Relationship between Coffee Consumption and Rheumatoid Arthritis

In the crude, four multiple logistic regression models, there was no significant association between coffee consumption and RA prevalence in both men and women (Table 3). After adjusting for all variables included in the study, compared to the no-coffee group, the ORs for RA in men drinking < 1 cup and 1–2 cups of coffee per day were 2.99 (95% CI 0.33–27.28) and 2.63 (95% CI 0.31–22.63), respectively. In women, the ORs for RA in the <1 cup, 1–2 cups, 2–3 cups, and ≥3 cups groups were 0.62 (95% CI 0.31–1.26), 0.67 (95% CI 0.33–1.37), 1.08 (95% CI 0.35–3.36), and 1.43 (CI 0.25–8.36), respectively. There were no men in the ≥2 cups group in our sample of RA patients.

## 4. Discussion

This study investigated the relationship between coffee consumption and RA, showing that coffee consumption was not related to the prevalence of RA in the Korean population. This is the first study on the relationship between coffee consumption and RA in the Asian population and considers various confounding factors, including smoking history, which critically affects the development of RA. Smoking adversely affects RA in various ways, including affecting the production of rheumatoid factor (RF), anti-citrullinated protein antibodies (ACPA), and causing oxidative stress [2]. Smoking is associated with ACPA production only in RA patients who carry the shared epitope as the human leukocyte antigen-DRB1-encoded 5-amino acid sequence motif [14]. However, there was no significant association between smoking status and RA prevalence, which may stem from the low prevalence of smoking amongst our female patients. Due to the cross-sectional design of the study, it suggested that the male participants diagnosed with RA might to have quit smoking. In addition, the possibility exists that there are fewer RA patients in Korean who carry the RA shared epitope. In this study, the higher the coffee consumption, the greater the number of cigarettes smoked, suggesting that Koreans usually smoke while drinking coffee. The lower the socioeconomic status (household income or education), the higher the prevalence of RA; however, the higher the socioeconomic status, the higher the coffee consumption. In addition, there were differences in the prevalence of chronic diseases (DM, HTN, and dyslipidemia) according to the degree of coffee consumption in this study, and these chronic diseases can affect the prevalence of RA [15]. Since there are various confounding factors between coffee consumption and the risk of RA, we adjusted for possible confounding factors in this study; however, other factors not included in this study may also have affected the associations between RA and coffee consumption.

Coffee contains more than 800 ingredients, with caffeine as the main compound. Caffeine acts as an antagonist to the A_1_ and A_2A_ subtypes of the four adenosine receptors (A_1_, A_2A_, A_2B_, and A_3_) [16]. Adenosine inhibits inflammation through these receptors and inhibits the production of cytokines, such as TNF-α, IL-1, and IL-6 [17,18]. In particular, A_2A_ receptors induce deactivation and apoptosis in T cells [18]. Therefore, caffeine may increase the risk of RA as a mechanism. In addition to caffeine, other major components of coffee, including cafestol, kahweol, chlorogenic acid, and furans, have anti-inflammatory and antioxidant properties [19,20]. Free radicals oxidize blood vessel walls, protein molecules, deoxyribonucleic acid (DNA), carbohydrates, and lipids, resulting in various human diseases [20]. RA has also been associated with oxidative stress. Oxidative stress increases the expression of pro-inflammatory cytokines and the production of RF, ACPA, and metalloproteinases, and causes damage to DNA, resulting in the development and progression of RA [21]. Unlike other studies, in this study, coffee consumption was not significantly associated with RA, and men who drank more than two cups, and women who drank three cups of coffee, did not show a greater likelihood of having RA. Since the contents and ingredients are different for each type of coffee bean, the anti-inflammatory and antioxidant effects may act differently. The most widely consumed coffees are Arabica and Robusta. Two-thirds of the coffee consumed in Korea was Arabica, and one-third was Robusta. Robusta coffee had a superior effect on lipid profile and adiponectin level to that of Arabica coffee [22], and lipid metabolism is related to inflammation, potentially explaining the absence of association between coffee and the high prevalence of RA in Korea, unlike previous observational studies in Western countries. In addition, residues of solvents (benzene, trichloroethylene, carbon tetrachloride, acetone, ammonium hydroxide, sulfuric acid, ethyl acetate, and methylene chloride) used in the process of extracting caffeine from coffee for decaffeinated coffee might have influenced the development of RA. Many of these solvents have been known to affect a number of connective tissue diseases, including RA, scleroderma, and systemic lupus erythematosus [23]. However, the effects of these solvents may have been minimized in our study because decaffeinated coffee is rarely consumed in Korea [24].

Mendelian randomization studies have shown that there was no causal relationship between coffee consumption and RA [25], which is consistent with our findings. Various factors such as coffee type, roasting method, and coffee drinking habits are related to health factors; thus, more detailed and controlled studies investigating coffee and health factors, in addition to one’s genes, are needed. Despite the difficulty in researching the type, amount, and habits of coffee consumption, this study has several strengths. This study evaluated the relationship between coffee consumption and RA in Korea by analyzing authoritative nationwide data. In addition, our results were sequentially adjusted for various possible confounding factors, including smoking, chronic diseases, and socioeconomic status.

This study also had some limitations. First, a causal relationship could not be determined because of the study’s cross-sectional design. Second, the definitions of RA and coffee consumption were based on self-reports. Consequently, these data may have been influenced by systematic errors in individuals’ consideration, which may have led to non-differential misclassification or overestimation. In particular, the presence of RF and ACPA may affects the diagnosis of RA and the relationship between RA and coffee [26], which has not been investigated in this study. Third, although this study classified coffee consumption based on the number of cups of coffee consumed per day, cup size, strength, and concentration of coffee may vary considerably. Fourth, there has been no investigation of other foods that may affect the risk of RA, such as fish, fruit, vegetables, and tea [27]. Finally, the small number of RA patients in our study may not have revealed the significant relationship; thus, a larger-scale study is needed. However, such a large-scale prospective study for investigating the relationship between coffee consumption and the prevalence of a disease is not realistic due to the difficulties in recruiting and retaining such a large number of participants.

## 5. Conclusions

The present study demonstrated that the prevalence of RA was not associated with coffee consumption in the general Korean population. This is the first Asian study on the relationship between coffee consumption and RA. However, since various factors affect the relationship between coffee consumption and RA, attention should be paid to interpretation, and further studies and intervention trials should be conducted.

## Figures and Tables

**Table 1:** Characteristics of participants with and without rheumatoid arthritis.

	Male (*n* = 4819)			Female (*n* = 7646)		*p*
	RA (*n* = 23)	Non-RA (*n* = 4796)	*p*	RA (*n* = 72)	Non-RA (*n* = 7574)	
Age (years)	56.57 ± 6.07	43.03 ± 12.80	0.000	53.07 ± 9.10	43.58 ± 12.38	0.000
BMI (kg/m^2^)	23.44 ± 2.47	24.56 ± 3.41	0.102	23.24 ± 3.59	23.17 ± 3.56	0.967
HTN			0.799			0.001
Normal	10 (43.5)	2009 (41.9)		32 (44.5)	4944 (65.3)	
Pre-HTN	6 (26.1)	1541 (32.1)		24 (33.3)	1411 (18.6)	
HTN	7 (30.4)	1246 (26.0)		16 (22.2)	1219 (16.1)	
DM			0.574			0.524
Normal	16 (69.6)	3037 (63.3)		57 (79.2)	5877 (77.6)	
IFG	4 (17.4)	1282 (26.7)		13 (18.1)	1252 (16.5)	
DM	3 (13.0)	477 (10.0)		2 (2.8)	445 (5.9)	
Dyslipidemia			0.391			0.811
No	12 (52.2)	2965 (61.8)		40 (55.6)	4345 (57.4)	
Yes	11 (47.8)	1831 (38.2)		32 (44.4)	3229 (42.6)	
Alcohol			0.123			0.529
Non-drinker	6 (26.1)	580 (12.1)		25 (34.7)	2181 (28.8)	
Moderate drinker	13 (56.5)	3201 (66.7)		43 (59.7)	4984 (65.8)	
Heavy drinker	4 (17.4)	1015 (21.2)		4 (5.6)	409 (5.4)	
Smoking			0.074			0.714
Non-smoker	3 (13.0)	1177 (24.5)		66 (91.6)	6709 (88.6)	
Former smoker	13 (56.5)	1640 (34.2)		3 (4.2)	437 (5.8)	
Current smoker	7 (30.5)	1979 (41.3)		3 (4.2)	428 (5.6)	
Income level			0.001			0.108
Lowest	7 (30.4)	397 (8.3)		12 (16.7)	743 (9.8)	
Lower middle	5 (21.7)	1110 (23.1)		19 (26.4)	1907 (25.2)	
Upper middle	2 (8.8)	1570 (32.7)		15 (20.8)	2374 (31.3)	
Highest	9 (39.1)	1719 (35.9)		26 (33.7)	2550 (33.7)	
Education level			0.000			0.000
Primary school or lower	6 (26.1)	328 (6.8)		22 (30.6)	881 (11.6)	
Middle school	4 (17.4)	373 (7.8)		13 (18.1)	728 (9.6)	
High school	4 (17.4)	1878 (39.2)		22 (30.6)	2941 (38.9)	
University or higher	9 (39.1)	2217 (46.2)		15 (20.7)	3024 (39.9)	
Daily coffee consumption			0.418			0.836
None	1 (4.3)	475 (9.9)		13 (18.1)	1023 (13.5)	
<1 cup	9 (39.1)	1478 (30.8)		25 (34.7)	2860 (37.8)	
1–2 cups	13 (56.6)	2341 (48.8)		26 (36.1)	2883 (38.1)	
2–3 cups	0 (0.0)	382 (8.0)		6 (8.3)	642 (8.6)	
≥3 cups	0 (0.0)	120 (2.5)		2 (2.8)	166 (2.2)	

Values are presented as mean ± standard deviation or number (%). RA, rheumatoid arthritis; BMI, body mass index; HTN, hypertension; DM, diabetes mellitus; IFG, impaired fasting glucose.

**Table 2:** Demographic characteristics of men (A) and women (B) according to coffee consumption.

(A)						
	Coffee Consumption (*n* = 4343)				No Coffee	*p*
	≥3 Cups (*n* = 120)	2–3 Cups (*n* = 382)	1–2 Cups (*n* = 2354)	<1 Cup (*n* = 1487)	(*n* = 476)	
Age (years)	39.85 ± 9.11	40.96 ± 11.18	46.00 ± 11.16	40.94 ± 14.27	37.97 ± 14.17	0.000
BMI (kg/m^2^)	25.04 ± 3.08	24.79 ± 3.29	24.65 ± 3.28	24.48 ± 3.61	24.05 ± 3.52	0.114
HTN						0.079
Normal	54 (45.0)	167 (43.7)	947 (40.2)	635 (42.7)	216 (45.4)	
Pre-HTN	36 (30.0)	117 (30.6)	760 (32.3)	469 (31.5)	165 (34.6)	
HTN	30 (25.0)	98 (25.7)	647 (27.5)	383 (25.8)	95 (20.0)	
DM						0.000
Normal	93 (77.5)	256 (67.0)	1403 (59.6)	962 (64.7)	339 (71.2)	
IFG	20 (16.7)	87 (22.8)	707 (30.0)	372 (25.0)	100 (21.0)	
DM	7 (5.8)	39 (10.2)	244 (10.4)	153 (10.3)	37 (7.8)	
Dyslipidemia						0.000
No	82 (68.3)	222 (58.1)	1375 (58.4)	980 (65.9)	318 (66.8)	
Yes	38 (31.7)	160 (41.9)	979 (41.6)	507 (34.1)	158 (33.2)	
Alcohol						0.000
Non-drinker	9 (10.6)	26 (6.8)	302 (12.8)	160 (10.8)	84 (17.6)	
Moderate drinker	46 (54.1)	279 (73.0)	1543 (65.5)	1019 (68.5)	288 (60.5)	
Heavy drinker	30 (35.3)	77 (20.2)	509 (21.6)	308 (20.7)	104 (21.8)	
Smoking						0.000
Non-smoker	26 (21.7)	78 (20.4)	395 (16.8)	467 (31.4)	214 (45.0)	
Former smoker	33 (27.5)	136 (35.6)	775 (32.9)	562 (37.8)	147 (30.8)	
Current smoker	61 (50.8)	168 (44.0)	1184 (50.3)	458 (30.8)	115 (24.2)	
Income level						0.000
Lowest	7 (5.8)	22 (5.8)	164 (7.0)	151 (10.2)	60 (12.6)	
Lower middle	22 (18.3)	70 (18.3)	575 (24.4)	337 (22.7)	111 (23.3)	
Upper middle	38 (31.7)	118 (30.9)	789 (33.5)	488 (32.8)	139 (29.2)	
Highest	53 (44.2)	172 (45.0)	826 (35.1)	511 (34.4)	166 (34.9)	
Education level						0.000
Primary school or lower	0 (0.0)	9 (2.4)	211 (9.0)	94 (6.3)	20 (4.2)	
Middle school	4 (3.4)	21 (5.4)	216 (9.2)	113 (7.7)	23 (4.8)	
High school	31 (25.8)	90 (23.6)	888 (37.7)	637 (42.8)	236 (49.6)	
University or higher	85 (70.8)	262 (68.6)	1039 (44.1)	643 (43.2)	197 (41.4)	
RA						0.418
No	120 (100.0)	382 (100.0)	2341 (99.4)	1478 (99.4)	475 (99.8)	
Yes	0 (0.0)	0 (0.0)	13 (0.6)	9 (0.6)	1 (0.2)	
**(B)**						
	**Coffee Consumption (*n* = 6610)**				**No Coffee**	***p***
	**≥3 Cups** **(*n* = 168)**	**2–3 Cups** **(*n* = 648)**	**1–2 Cups** **(*n* = 2909)**	**<1 Cup** **(*n* = 2885)**	**(*n* = 1036)**	
Age (years)	39.32 ± 9.91	40.92 ± 10.42	44.70 ± 10.92	44.07 ± 13.19	42.06 ± 14.67	0.000
BMI (kg/m^2^)	22.71 ± 3.66	22.99 ± 3.56	23.42 ± 3.49	23.23 ± 3.62	22.53 ± 3.42	0.000
HTN						0.000
Normal	123 (73.2)	467 (72.1)	1863 (64.0)	1821 (63.1)	702 (67.8)	
Pre-HTN	31 (18.5)	113 (17.4)	561 (19.3)	540 (18.7)	190 (18.36)	
HTN	14 (8.3)	68 (10.5)	485 (16.7)	524 (18.2)	144 (11.7)	
DM						0.001
Normal	135 (80.4)	537 (82.9)	2251 (77.42)	2207 (76.5)	804 (77.6)	
IFG	27 (16.1)	91 (14.0)	503 (17.3)	489 (16.9)	155 (15.0)	
DM	6 (3.6)	20 (3.1)	155 (5.3)	189 (6.6)	77 (7.4)	
Dyslipidemia						0.000
No	123 (73.2)	414 (63.9)	1639 (56.3)	1626 (56.42)	583 (56.3)	
Yes	45 (26.8)	234 (36.1)	1270 (43.7)	1259 (43.6)	453 (43.7)	
Alcohol						0.000
Non-drinker	24 (14.3)	136 (21.0)	709 (24.4)	872 (30.2)	465 (44.9)	
Moderate drinker	130 (77.4)	472 (72.8)	2010 (69.1)	1890 (65.5)	525 (50.7)	
Heavy drinker	14 (8.3)	40 (6.2)	190 (6.5)	123 (4.3)	46 (4.4)	
Smoking						0.000
Non-smoker	127 (75.6)	550 (84.9)	2535 (87.1)	2628 (91.1)	935 (90.3)	
Former smoker	18 (10.7)	46 (7.1)	161 (5.5)	156 (5.4)	59 (5.7)	
Current smoker	23 (13.7)	52 (8.0)	213 (7.3)	101 (3.5)	42 (4.1)	
Income level						0.000
Lowest	2 (1.2)	34 (5.2)	278 (9.6)	316 (11.0)	125 (12.1)	
Lower middle	27 (16.1)	106 (16.4)	729 (25.1)	760 (26.3)	304 (29.3)	
Upper middle	45 (26.7)	183 (28.2)	923 (31.7)	924 (32.0)	314 (30.3)	
Highest	94 (56.0)	325 (50.2)	979 (33.6)	885 (30.7)	293 (28.3)	
Education level						0.000
Primary school or lower	3 (1.8)	19 (2.9)	319 (11.0)	414 (14.4)	148 (14.3)	
Middle school	10 (6.0)	25 (3.9)	304 (10.5)	296 (10.3)	106 (10.2)	
High school	45 (26.9)	211 (32.6)	1166 (40.0)	1127 (39.0)	414 (40.0)	
University or higher	110 (65.5)	393 (60.3)	1120 (38.5)	1048 (36.3)	368 (35.5)	
RA						0.836
No	166 (98.8)	642 (99.1)	2883 (99.1)	2860 (99.1)	1023 (98.7)	
Yes	2 (1.2)	6 (0.9)	26 (0.9)	25 (0.9)	13 (1.3)	

Values are presented as mean ± standard deviation or number (%). BMI, body mass index; HTN, hypertension; DM, diabetes mellitus; IFG, impaired fasting glucose. RA, rheumatoid arthritis.

**Table 3:** ORs and 95% CIs for RA by coffee consumption in men (A) and women (B).

(A)									
	None	<1 Cup		1–2 Cups		2–3 Cups		≥3 Cups	
	OR	OR	95% CI	OR	95% CI	OR	95% CI	OR	95% CI
Crude	1.00	2.89	0.37–22.89	2.64	0.34–20.21	N/A	N/A	N/A	N/A
Model I	1.00	2.30	0.29–18.35	1.90	0.25–14.72	N/A	N/A	N/A	N/A
Model II	1.00	2.40	0.30–19.37	1.93	0.25–15.06	N/A	N/A	N/A	N/A
Model III	1.00	2.78	0.33–23.57	1.99	0.24–16.69	N/A	N/A	N/A	N/A
Model IV	1.00	2.99	0.33–27.28	2.63	0.31–22.63	N/A	N/A	N/A	N/A
**(B)**									
	**None**	**<1 Cup**		**1–2 Cups**		**2–3 Cups**		**≥3 Cups**	
	**OR**	**OR**	**95% CI**	**OR**	**95% CI**	**OR**	**95% CI**	**OR**	**95% CI**
Crude	1.00	0.69	0.35–1.35	0.71	0.36–1.39	0.74	0.28–1.95	0.95	0.21–4.24
Model I	1.00	0.71	0.36–1.40	0.73	0.37–1.44	1.02	0.37–2.80	1.40	0.30–6.58
Model II	1.00	0.68	0.34–1.36	0.69	0.34–1.37	0.98	0.36–2.72	1.41	0.29–6.78
Model III	1.00	0.62	0.30–1.24	0.65	0.32–1.33	0.92	0.33–2.61	1.34	0.27–6.68
Model IV	1.00	0.62	0.31–1.26	0.67	0.33–1.37	1.08	0.35–3.36	1.43	0.25–8.36

Model I: adjusted for age and BMI. Model II: adjusted for age, BMI, HTN, DM, and dyslipidemia. Model III: adjusted for age, BMI, HTN, DM, dyslipidemia, alcohol consumption, and smoking status. Model IV: adjusted for age, BMI, HTN, DM, dyslipidemia, alcohol consumption, smoking status, and socioeconomic status. OR, odds ratio; CI, confidence interval; RA, rheumatoid arthritis; BMI, body mass index; HTN, hypertension; DM, diabetes mellitus.

## Data Availability

All National Health and Nutrition Examination Survey files are available from the Korea Centers for Disease Control and Prevention database (URL https://knhanes.cdc.go.kr/knhanes/sub03/sub03_02_02.do, accessed on 18 September 2020).
